# How activated NLRs induce anti-microbial defenses in plants

**DOI:** 10.1042/BST20210242

**Published:** 2021-10-08

**Authors:** Farid El Kasmi

**Affiliations:** Center for Plant Molecular Biology (ZMBP), University of Tübingen, Tübingen Germany

**Keywords:** coiled-coil NLR (CNL), effector-triggered immunity, helper NLRs (RNLs), NADase, plant immunity, resistosome

## Abstract

Plants utilize cell-surface localized and intracellular leucine-rich repeat (LRR) immune receptors to detect pathogens and to activate defense responses, including transcriptional reprogramming and the initiation of a form of programmed cell death of infected cells. Cell death initiation is mainly associated with the activation of nucleotide-binding LRR receptors (NLRs). NLRs recognize the presence or cellular activity of pathogen-derived virulence proteins, so-called effectors. Effector-dependent NLR activation leads to the formation of higher order oligomeric complexes, termed resistosomes. Resistosomes can either form potential calcium-permeable cation channels at cellular membranes and initiate calcium influxes resulting in activation of immunity and cell death or function as NADases whose activity is needed for the activation of downstream immune signaling components, depending on the N-terminal domain of the NLR protein. In this mini-review, the current knowledge on the mechanisms of NLR-mediated cell death and resistance pathways during plant immunity is discussed.

## Introduction

Beside physical barriers, including bark, leaf hairs (trichomes), waxy cuticles and plant cell walls, plant-invading (micro-)organisms have to face a highly sophisticated and heavily interconnected two-tiered, receptor based innate immune system [1]. At the cell surface, plasma membrane spanning pattern-recognition receptors (PRRs) perceive the presence of invading organisms, like insects, fungi, oomycetes, bacteria or parasitic plants, by sensing so-called microbe- or host-derived damage-associated molecular patterns (MAMPs/DAMPs) [2,3]. PRRs are receptor kinases (RKs) or receptor-like proteins (RLPs), and MAMP/DAMP perception leads to a cascade of immune responses, commonly referred to as pattern-triggered immunity (PTI) [3]. PTI induces a rapid calcium (Ca^2+^) influx, production of reactive oxygen species (ROS), the release of anti-microbial molecules and proteins into the apoplast (the intercellular space), initiation of mitogen-activated protein kinase (MAPK) signaling, transcriptional reprogramming and the up-regulation of defense genes, among others [3,4]. This ‘cocktail’ of induced responses is often sufficient to successfully defend host non-adapted microbes and, to a limited extent, host-adapted pathogens. To interfere with PTI and to counter resistance, thus promote pathogenesis, adapted pathogens evolved effector molecules and proteins that they secrete into host cells or the apoplast [5,6].

However, in resistant plants, effectors can be perceived either directly or indirectly by a second class of immune receptors, the intracellular nucleotide-binding (NB) leucine-rich repeat (LRR) receptor (NLR) family. NLR activation through effector recognition initiates a robust immune response referred to as effector-triggered immunity (ETI) [7,8]. ETI responses are both similar and distinct in their molecular signature to PTI responses [9,10]. Initiation of ETI by activated NLRs triggers a prolonged calcium influx [11]and often results in the induction of the hypersensitive response (HR), a form of programmed cell death at the site of infection that further restricts pathogen proliferation and spread [12]. NLR-induced HR triggers the establishment of systemic acquired resistance (SAR), which primes systemic tissues to faster and more robustly respond to secondary infections [13]. It is important to emphasize that during natural infections ETI is often, if not always, happening in the presence of activated PTI — this is also the case in most experimental set-ups used to study or assay for ETI. Exceptions may be the use of transgenic plants expressing pathogenic effectors in an inducible fashion [9,10], or during viral infections — although more and more evidences are being presented that suggest that there is a PTI(-like) activation during viral infections [14–16].

NLR-like proteins can also be found in animals and fungi, where they convergently evolved as intracellular surveillance proteins to detect different kinds of pathogens and to function in allorecognition, respectively [7,8,17]. The first plant *NLR* genes were cloned in the early 1990s from tobacco (*Nicotiana tabacum*; [18]) and *Arabidopsis thaliana* (hereafter: Arabidopsis; [19,20]), and since then, our knowledge of how these important proteins contribute to immunity has tremendously expanded [21]. Especially research made in the last couple of years has enabled the field to make a huge step forward in understanding NLR activation, function in cell death initiation and immune signaling. Intriguingly, these results demonstrate that the activation of at least some NLRs results in oligomerization, which enables NLRs to form either an active holoenzyme or a potential cation channel at cellular membranes, depending on their N-terminal domain architecture.

This mini-review aims at summarizing some milestones in NLR biology. It further provides an overview of the recent achievements in determining how NLRs might mobilize antimicrobial defenses, while simultaneously it outlines the still unanswered questions of NLR-mediated immunity.

## NLR domains and structures

Most plant NLRs, as well as the metazoan NLRs, have a modular and conserved tripartite domain architecture, with a variable N-terminal domain, a central nucleotide-binding and oligomerization domain (NB or NOD), and a C-terminal leucine-rich repeat (LRR) domain. The plant NB domain (also referred to as NB-ARC, nucleotide-binding adaptor shared by apoptotic protease activating factor-1 (Apaf-1), Resistance (R)-protein, and cell death abnormal (CED) protein) is subdivided into three subdomains: the NB domain, an ARC1 or helical domain 1 (HD1) and an ARC2 or winged-helix domain (WHD), and belongs to the AAA+ ATPase superfamily [22,23]. The variable N-terminal domain can either be a Toll/Interleukin-1 receptor/Resistance protein (TIR) domain, a coiled-coil (CC) or a resistance to powdery mildew 8 (RPW8)-like CC (CC_R_) domain [24]. The different NLR classes are therefore also termed as TNLs (with a TIR domain), CNLs (with a CC domain) and RNLs (harboring a CC_R_ domain). The CC and CC_R_ domains share structural homology with the N-terminal 4-helical bundle of mammalian and plant mixed-linage kinase like (MLKL) proteins and pore-forming fungal HET-s/Helo domain containing proteins [24–29]. TIR domains have been shown to be structurally and functionally conserved — at least to some extent — and can be found in prokaryotic and other eukaryotic organisms [30,31], where TIR domain-containing proteins have also been implicated in immunity and initiation of cell death responses [32–34]. Plant NLR LRR domains have the classical horseshoe shaped structure with parallel beta-strands facing the concave (inner) side and on the convex side with variable secondary structures, including alpha helical regions [35].

Intramolecular interactions between the different domains keep NLR proteins in an inactive, but signaling competent state, in which the NB-ARC domain binds adenosine diphosphate (ADP) via conserved and functionally important motifs. The NB-ARC domain likely functions as a molecular switch, transitioning the NLR from an inactive ADP-bound to an active adenosine triphosphate (ATP)-bound state [23,36,37]. However, ATP-binding to the NB-ARC domain might not always be associated with NLR activity, as recently shown for the Arabidopsis TNL Recognition of *Peronospora parasitica* 1A (RPP1) [38]. The C-terminal LRR domain often plays an important role in the autoinhibitory process via interactions with the NB-ARC and/or the N-terminal domains [39]. Deletion of the LRR domain can, for example, result in the constitutive activation of NLRs in both plants and animals [40–42]. In addition to this regulatory function, many LRR domains or C-terminal LRR-adjacent domains (C-JID) are also involved in direct binding of effectors [38,43]. Cryogenic electron microscopy (cryo-EM) analyses have revealed that the LRR domain together with C-CJID domain of two TNLs, Recognition of XopQ1 (ROQ1) and RPP1A, is required for direct effector recognition [38,43]. Furthermore, a recent survey of NLR complements of over 60 Arabidopsis ecotypes and more than 50 *Brachypodium distachyon* lines confirmed that LRR domains of many NLRs show high allelic diversity especially in residues or regions predicted to be specificity determining [44]. This suggests that the LRR modularity functions to generate binding sites to non-self as well as self-molecules, and thus NLRs containing such LRR domains might be a source for new direct and indirect effector-binding/sensing immune receptors [44]. The N-terminal CC/CC_R_ and TIR domains mediate NLR signaling and are required or at least participate in NLR oligomerization, and can therefore be considered as the signaling domains. A number of studies demonstrated that overexpression of isolated CC/CC_R_ or TIR domains was sufficient to induce cell death or auto-immunity [28,39,45–48], further supporting their signaling function.

## Effector recognition and NLR activation

Within the last two years the publication of cryo-EM structures of three full length plant NLRs, the Arabidopsis CNL ZAR1 [23,49] and TNL RPP1A [38], and the *N. benthamiana* TNL ROQ1[43], have been released, which enabled us to better understand the molecular events downstream of effector recognition that lead to NLR activation. NLR proteins perceive the presence or activity of effectors either through direct interaction or by sensing modifications of host effector targets or decoys of such [50]. Direct effector recognition is often mediated by the LRR domain, a C-terminal extension termed the C-terminal jelly roll and Ig-like domain (C-JID) or Post-LRR (PL) domain or through a non-canonical ‘integrated domain’ (ID) that can often be found at the C-terminal end, but can also be integrated somewhere else in the protein domain structure [38,43,51–53]. In a recent study, structure-guided engineering was used to expand the recognition profile of the rice NLR Pikp-1 to variants of a rice blast pathogen effector by mutating residues important for effector binding in the ID of Pikp-1 [54]. This clearly demonstrates how structure-function analysis enable us to understand effector recognition and how this knowledge can be directly applied to generate (modified) improved immune receptors for agriculture.

Indirect recognition of effectors is mediated by NLRs that guard effector targets or that interact inducibly with effector-modified host proteins. Often these host proteins have a function in immunity, and one of the best-studied examples is the small Arabidopsis protein RPM1-interacting protein 4 (RIN4), which is guarded by two CNLs RPM1 and RPS2 [55–57]. RIN4 is targeted by numerous bacterial effectors which post translationally modify specific RIN4 residues or proteolytically cleave the protein [55,57–60]. It is believed that these modifications lead to conformational changes in the preformed NLR-guardee complex that than result in NLR activation and downstream signaling. How the different modifications on RIN4 activate RPM1 or RPS2 in detail requires further analysis. Given that activation of the two CNLs require different modifications on RIN4 it can be assumed that the molecular mechanism(s) of activation might vary as well. Thus, how the conformational change allowing the exchange of ADP by ATP and the oligomerization is induced may differ, but the outcome, formation of a functional ‘resistosome’, may be similar.

**Figure 1.**
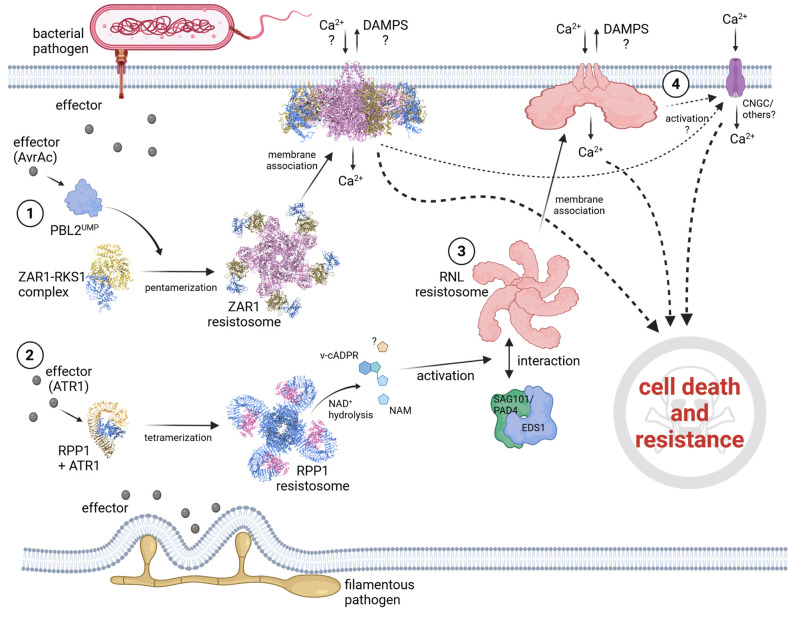
NLR mediated immune signaling. Schematic presentation of CNL (Arabidopsis ZAR1 (**1**)), TNL (Arabidopsis RPP1 (**2**)) and helper NLR (Arabidopsis RNL NRG1.1 (**3**)) cell death and resistance signaling. (**1**) The bacterial effector AvrAc uridylates the receptor-like cytoplasmic kinase (RLCK) PBL2 resulting in PBL2^UMP^. PBL2^UMP^ binds to the preformed ZAR1–RKS1 complex via interaction with RKS1, which induces a conformational change in the ZAR1–RKS1 complex resulting in nucleotide exchange (ADP to ATP) in the ZAR1 nucleotide-binding domain (NB-ARC) and eventually to the pentamerization of this tetrameric ZAR1–RKS1–PBL2^UMP^ complex into a so-called resistosome. The ZAR1 resistosome relocalizes to the plasma membrane and forms a calcium permeable cation channel via the N-terminal coiled-coil (CC) domains of the ZAR1 proteins [61,63]. (**2**) RPP1 recognizes and directly binds the oomycete-derived effector ATR1 via its leucine-rich repeat domain and the C-terminal jelly roll and Ig-like domain (C-JID). ATR1 binding induces the tetramerization of four RPP1-ATR1 sub-complexes into a RPP1 resistosome and thus a conformational activation or RPP1. Interactions between the RPP1 NB-ARC domains and the N-terminal TIR domains stabilize the resistosome complex without the necessity of ATP binding. RPP1 tetramerization results in the opening up of the NADase active site, transforming the TIR domains into an active holoenzyme [38]. Hydrolysis of NAD+ results in the production of various potential signaling molecules, such as variant cyclic ADPR (v-cADPR), that are hypothesized to activate and induce interaction of the TNL downstream signaling components EDS1, PAD4, SAG101 and the RNLs (helper NLRs). (**3**) How the EDS1/PAD4 or EDS1/SAG101 RNL components are activated by TIR domain NADase activity is not clear. Recently, it was shown that the Arabidopsis NRG1.1/NRG1A and ADR1 (both RNLs) can form activation-dependent oligomers and function also as calcium permeable cation channels at the plasma membrane [27,64]. Note: There is no experimental evidence yet that RNL resistosomes form ring-like structures (here a hexamere), which are similar to the pentameric ZAR1 ring-like resistosome. (**4**) It is known that NLR-activated effector-triggered immunity leads to calcium influx, which is required for cell death and resistance signaling, but how channel or pore formation by ZAR1 or NRG1.1/NRG1A is causing cell death (cell collapse) is not understood. It is possible that the calcium influx activates other cation channels important for immunity (for example the CNGCs) and components required for cell collapse, and that damage-associated molecular patterns (DAMPs) might also be released by the NLR formed pores to create signals enhancing immune responses that may lead to systemic resistance and maybe cell death. Created with BioRender.com.

How effector-recognition can mechanistically induce NLR activation was impressively demonstrated by recent cryo-EM studies that presented the structure of inactive and effector-activated Arabidopsis CNL ZAR1 [23,49,61]. ZAR1 can detect the presence/function of a large number of effectors from different pathogens [62]. Inactive ZAR1 is bound to ADP and exists in a monomeric state, where it can interact with various receptor-like cytoplasmic kinases (RLCKs) of the RLCK-XII subfamily. ZAR1-interacting RLCKs can be considered as ‘adaptor proteins’ in this complex, because they function as the docking-site for another subfamily of RLCKs (subfamily RLCK-VII), which, upon modifications by ZAR1-recognized effectors, bind to the preformed ZAR1–RLCK-XII complex. Interaction of the RLCK-VII member with the ZAR1–RLCK-XII complex removes a structural inhibition on the ZAR1 NB-ARC domain, which was exerted by parts of the RLCK-XII protein, and in turn leads to the exchange of ADP by ATP and eventually to oligomerization of the tertiary complexes into a so-called resistosome [49](Figure 1). Comparisons between the cryo-EM structure of inactive and active ZAR1 uncovered the mechanism of ZAR1 activation in fantastic detail and suggests a dynamic structural change from an inactive, to an intermediate and further into an active, signaling competent pentameric complex [23,61].

An activation mechanism similarly depending on effector recognition and oligomerization (specifically tetramerization) was recently postulated for the two plant TNLs RPP1A and ROQ1 [38,43]. Cryo-EM structures of effector-bound ROQ1 and RPP1A defined a tetramerization into a symmetric dimer of two dimers that was essential for cell death activity and immune signaling [38,43] (Figure 1). Complex formation, self-association and also heterooligomerization was shown to be important for the activity of many other NLRs, including CNLs, TNLs and the RNLs [27,28,48,65–69]. Interestingly, many plant NLRs already self-associate in the absence of effector-recognition — thus, in the pre-activation state (El Kasmi lab unpublished results and [48,64,65,68,70]). In these cases, sensing of the effector most likely induces conformational changes in the preformed dimers or oligomers allowing nucleotide exchange and possibly the interaction with downstream signaling components or the formation of resistosome-like complexes. It would be interesting to see whether for such NLRs a shift into high-molecular weight complexes could be observed upon activation, which would suggest a specific recruitment of downstream signaling components or the formation of higher oligomeric complexes.

## Immune signaling by activated NLRs — resistosomes as pores or holoenzymes

Plant NLR activation by effectors or by mutations affecting nucleotide binding leads to oligomerization and the formation of large NLR complexes, termed resistosomes [27,36,38,43,61]. Regardless of the type of NLR (TNL, CNL or CC_R_-NLR) oligomerization is most likely required to bring the N-terminal domains into close proximity so that these can facilitate their signaling function or the recruitment of downstream components. This is further supported by many reports demonstrating that the expression of TIR or CC/CC_R_ domains alone is sufficient to induce cell death and cause (auto-) immunity [28,39,45–48].

The mechanism(s) of initiating immunity is however very different between the two major NLR classes, the TNLs and CNLs. Intriguingly, all tested TNLs require the presence of members of the RNL family, a CC-NLR subfamily, for proper cell death and resistance activation, and therefore RNLs are also considered as helper NLRs [25,71–77]. In solanaceous plants (for example potato, tomato and tobacco) another family of helper NLRs was described, the NLR-required for cell death (NRC) proteins [78]. NRCs are typical CNLs and form a functional NLR network with phylogenetically related CNLs, and this NLR network most likely enhances the robustness and complexity of NLR-mediated immunity [79]. CNL function therefore can depend on helper NLRs but many CNLs are thought to function independent of helper NLR assistance — at least to a certain degree [71,73,76] — and these CNLs are also termed as ‘helper-independent’ NLRs or singletons [80].

## CNL signaling

The cryo-EM structure of activated full-length Arabidopsis CNL ZAR1 suggested that the CC domains of the five ZAR1 proteins in the resistosome complex form a funnel-like structure that may penetrate the plasma membrane to either build a pore or channel or to disrupt membrane integrity [61]. A recent follow-up study impressively demonstrated that the activated ZAR1 resistosome indeed forms a Ca^2+^ permeable cation channel at the plant plasma membrane [63]. Formation of this channel requires ZAR1 activation, oligomerization, (plasma) membrane localization and the formation of a pore by the CC domain. Specific negatively charged glutamate (E) residues of the CC domain positioned in the channel pore are required for cation (Ca^2+^) fluxes and the subsequent initiation of cell death and immunity. How ZAR1 activation exactly triggers cell death during an ETI response is still unclear. However, cell biological time-lapse analyses have shown that ZAR1 activation leads to intracellular calcium influx and reactive oxygen species (ROS) production, changes in morphology of cellular compartments/organelles and ultimately to rupture of the cell, most likely due to loss of plasma membrane integrity. All these responses were lost when the conserved glutamate (E11) was mutated, indicating that channel activity and therefore most likely Ca^2+^ influxes are upstream of these responses [63].

It was previously shown that the Arabidopsis CNL (RPM1) and the ADR1 RNL-subfamily self-associate and localize to and function at the plasma membrane to induce a cell death response [64,65]. Self-association of the ADR1s seems to be enhanced in an activation-dependent manner [64,81]. Another recent study demonstrates that autoactive forms of the Arabidopsis helper NLRs NRG1.1/NRG1A and ADR1, both members of the RNL subfamily, do also form Ca^2+^ permeable cation channels at the plasma membrane (Figure 1), and that RNL-mediated cell death and Ca^2+^ fluxes also required negatively charged residues in their CC_R_ domain [27]. The important negatively charged residues (E11 in ZAR1, D11 in ADR1 and E14 in NRG1.1/NRG1A) are not conserved in all CNLs, and thus there might be other mechanisms in place [82]. It is very likely that NRG1.1/NRG1A oligomerizes and forms high molecular weight complexes in an activation dependent manner — similar to ZAR1- as an NRG1.1/NRG1A P-loop loss-of-function mutant, affected in nucleotide binding, did not. Based on these recent findings and the fact that NLR-triggered ETI leads to prolonged calcium influxes, conceivably, all CNL and RNL triggered cell death responses may require the formation of some kind of channel, most likely cation channels, in cellular membranes. A dynamic, activation-dependent membrane localization was recently reported for Arabidopsis RNLs and a solanaceous NRC member, NRC4 [27,64,83], suggesting that probably all cell-death and resistance executing CNLs, including the RNLs and NRCs, function at a cellular membrane. This assumption is supported by several reports showing the inhibition of CNL-mediated cell death responses by general calcium channel blockers, for example lanthanum or gadolinium [11,27,63,65,84]. However, whether cation (specifically Ca^2+^) channel activity is also required for CNL and RNL-mediated signaling leading to disease resistance needs to be determined. There are some examples where NLR-mediated disease resistance does not require cell death or where cell death can occur even in the absence of resistance — thus, cell death and resistance are not necessarily coupled [12,85,86]. The observation that inhibition of Ca^2+^ fluxes by lanthanum blocks the Arabidopsis RPM1 (CNL)-triggered cell death response, but not disease resistance, may indicate that Ca^2+^ fluxes are, at least in case of RPM1 mediated immunity, not required for disease resistance [11,65]. However, the exact mechanism by which lanthanum is suppressing cell death initiated by NLRs is not known. Similarly, how long the lanthanum effect is lasting *in planta* is also not really studied, but this would be important to know, since determining resistance to infections in plants is normally a matter of days.

## TNL signaling

TNL-triggered immune responses require the presence of the helper NLRs (in Arabidopsis the RNL members of the ADR1 and NRG1 subfamilies) and the lipase-like protein family members Enhanced Disease Susceptibility 1 (EDS1), Phytoalexin Deficient 4 (PAD4) and Senescence-Associated Gene 101 (SAG101) [25,71,77,81,87–89] (Figure 1). Effector-recognition by TNLs induces self-association and in case of TNL-pairs, for example the Arabidopsis RPS4 and RRS1 proteins, hetero-association that is required for proper immune signaling [48]. The formation of a tetrameric TNL-resistosome by effector-bound RPP1 or ROQ1 enables their TIR domains to build a twofold symmetric dimer of TIR domain dimers, which is essential for the recently discovered nicotinamide adenine dinucleotide (NAD+) hydrolase (NADase) activity of this domains [38,43,90,91] (Figure 1). Plant TIR domain NADase activity depends on the presence of a highly conserved glutamate residue that can also be found in TIR domains of prokaryotes and other eukaryotes [30,34], for example in the mammalian TIR domain containing sterile alpha and TIR motif containing 1 (SARM1) protein, which mediates axonal cell death via its NADase enzymatic activity [92,93]. NAD+ and its cleavage products are important for many essential cellular functions, including immune signaling and the activation of calcium channels [34]. The enzymatic hydrolysis of NAD+ by SARM1 leads to NAD+ depletion, which is hypothesized to be sufficient for SARM1 induced cell death [93]. However, a detectable NAD+ depletion by plant TIRs could not be observed [90]. Plant TIR domains hydrolyze NAD+ into ADP-Ribose (ADPR), Nicotinamide (NAM) and variant cyclic-ADPR (v-cADPR) [90,91], whereas SARM1 activity generates NAM, ADPR and cADPR [92], thus plant and animal catalytic products differ from each other. Intriguingly, heterologous expression of SARM1 TIR domain in plant cells also induces a cell death response, similar to overexpression of plant TIR domains [90,91]. However, the SARM1 TIR domain cell death activity in plants does not depend on EDS1 [90,91] and most likely also not on the RNL helper NLRs, although both components are absolutely required for plant TIR-induced cell death [77]. This indicates that the mechanism of cell death induction mediated by SARM1 and plant TIR domains is different and/or that v-cADPR produced by plant TIR domains is required for signal initiation upstream of EDS1 and RNLs. It is worth to mention that in planta expression of bacterial TIR domains, which also produce a v-cADPR, was also not sufficient to activate a cell death response [94]. Probably because the v-cADPR produced differs or bacterial TIR domains are not able to interact with other proteins required for plant TIR domain signaling. However, the exact mechanism of how plant TIR domain NADase-activity and the thereby produced signaling molecules initiate or activate immune responses is largely unknown.

## NLR-mediated mobilization of antimicrobial defenses in ETI — the great unknown or just an unrestrained re-installment of PTI!

CNL-mediated ETI and also TNL-mediated ETI, which is essentially activation of RNLs [71,73–76] (Figure 1), lead to measurable increases in cytosolic calcium concentrations, the initiation of ROS production and activation of MAPK as well as calcium-dependent protein kinase (CDPK) signaling [95,96]. These immune outputs are all responses that are shared with PTI [97–99]. One difference, also this is not exclusively true for all cases, is the initiation of the HR cell death at the final stage of ETI. HR cell death is often associated to be specific for NLR activity, although many studies reported that perception of apoplastic effectors or even MAMPs by PRRs does also induce HR-like cell death responses [100–103]. Furthermore, overexpression of or loss-of-function mutations in important plant PRR co-receptors, like the LRR-RK SOBIR1 or members of the SERK family (for example BAK1 or BKK1), induces severe autoimmune phenotypes with obvious cell death symptoms [104–110]. However, recent findings demonstrated that some of these autoimmune phenotypes induced by a disturbed LRR-RK abundancy are indeed linked to NLR activity [111,112]. Similarly, resistance and cell death triggered by perception of extracellular effectors or ligands by PRRs of the LRR-RLP type is also reported to require helper NLR (RNL) presence [79,113–116]. Another recent finding suggests that signaling by TNLs is contributing to full PTI responses and that PTI induces the rapid transcriptional up-regulation of many TNL genes [117]. Therefore, immunity induced by PRR activation may always depend on an actual NLR activation and thus, is feeding into the classical ETI pathway components [116]. The already very fine line separating PTI and ETI is even further being blurred by two recent and important studies demonstrating a mutual potentiation and interdependency of PTI and ETI pathways [9,10], suggesting convergence of immune signaling initiated by the two receptor-based tiers of plant immunity. The transcriptional activation of important PTI pathway genes and the induced phosphorylation of many essential immune components upon NLR activation is further supporting the idea of a shared or common regulatory or signaling node for cell surface and intracellular immune receptors [97,118,119]. It will be interesting to see, whether such a ‘PTI-pathway’ activation can also be observed in NLR autoimmune mutants or during hybrid incompatibility [68,69,120,121].

The mechanisms leading to the loss of cell integrity during ETI are not well studied. There are a few reports showing that NLR activation or the expression of viral, bacterial and fungal effectors can induce severe morphological cellular phenotypes, like the fusion of the vacuole with the plasma membrane [122], alterations of vacuolar structures (El Kasmi lab unpublished; [63]), the expansion of the nucleus [123], chloroplast clustering around the nucleus [124], cytoplasmic shrinkage, mitochondrial swelling and many more [125]. It is likely that these cellular changes and the loss of plasma membrane integrity is leading to a release or (calcium-) induced production of DAMPs [126,127], molecular signals that are known to be potent immune activators [128]. Production of systemic signals is indeed known to take place during both PTI and ETI to induce priming and a systemic acquired resistance [13,129–131]. However, whether these signals are released prior to cell death and if yes, how they are released from the infected cells is still unclear. One attractive possibility would be that activated CNLs and RNLs could, in addition to being calcium permeable channels or pores, allow the release of such DAMP molecules even before the actual rupture or breakdown of the infected cell. This would be similar to the function of pore forming and cell death inducing proteins, for example gasdermin or MLKL in the animal kingdom, or Het-S/HeLo like proteins in fungal organisms [132–134]. Plants do also posses MLKL like proteins capable of inducing cell death and involved in TNL-mediated resistance signaling [26]. It will be interesting to see whether and how MLKL proteins and NLRs cooperate during plant immunity and if there is any kind of specificity.

NLR activation leads to calcium influxes and probably initiation of other ion fluxes, and this in turn can activate other immune regulatory proteins, including cyclic nucleotide-gated cation channels (CNGCs) [135] (Figure 1), CPKs and transcriptional regulators of the calmodulin-binding protein family that are crucial for expression of salicylic acid biosynthesis genes and genes important for production and release of antimicrobial molecules [95,136].

## Perspectives

- *Importance of the field*: Intracellular NLR receptors are important components of a plants defense system against host-adapted and non-adapted pathogens. NLR-triggered immunity leads to strong and robust defense responses and thus understanding their mechanism(s) of action is of high importance for agricultural research to generate better and durable resistant crop plants.
- *Current thinking*: Recognition of pathogen-derived effectors activates NLR proteins and induces their self-association, which in turn leads to oligomerization of their N-termini and the production of signaling molecules or the formation of a cation channel at cellular membranes, depending on the architecture of the N-terminal domain. NLR activity generally, but not always, leads to a strong immune response in the whole organism and an induced cell death of the infected cell.
- *Future directions*: Identifying NLR binding partners that might contribute to their specific functions and regulate their activities, will be crucial in enhancing our understanding of how the different types of NLRs mediate immunity against a wide variety of pathogens. Determining the exact site of NLR action and the mechanism of cell death induction is also one of the fields next important and interesting endeavors.

## Abbreviations

ADP: adenosine diphosphate
ATP: adenosine triphosphate
CNGCs: cyclic nucleotide-gated cation channels
DAMPs: damage-associated molecular patterns
EDS1: enhanced disease susceptibility 1
ETI: effector-triggered immunity
HR: hypersensitive response
ID: integrated domain
LRR: leucine-rich repeat
MAPK: mitogen-activated protein kinase
MLKL: mixed-linage kinase like
NAM: Nicotinamide
NB: nucleotide-binding
NLRs: nucleotide-binding LRR receptors
NRC: NLR-required for cell death
PAD4: Phytoalexin Deficient 4
PRRs: pattern-recognition receptors
PTI: pattern-triggered immunity
RIN4: RPM1-interacting protein 4
RPP1: Recognition of *Peronospora parasitica* 1A
SARM1: sterile alpha and TIR motif containing 1
TIR: Toll/Interleukin-1 receptor/Resistance protein
